# Effects of *NtSPS1* Overexpression on Solanesol Content, Plant Growth, Photosynthesis, and Metabolome of *Nicotiana tabacum*

**DOI:** 10.3390/plants9040518

**Published:** 2020-04-17

**Authors:** Ning Yan, Xiaolei Gai, Lin Xue, Yongmei Du, John Shi, Yanhua Liu

**Affiliations:** 1 Tobacco Research Institute of Chinese Academy of Agricultural Sciences, Qingdao 266101, China; duyongmei@caas.cn; 2 Yunnan Tobacco Leaf Company, Kunming 650000, China; gaixiaolei2019@163.com; 3 Anhui Wannan Tobacco Leaf Co., Ltd., Xuancheng 242000, China; xuelin-xx@163.com; 4 Guelph Food Research Center, Agriculture and Agri-Food Canada, Guelph, ON N1G 5C9, Canada; john.shi@agr.gc.ca

**Keywords:** *Nicotiana tabacum*, solanesol, solanesyl diphosphate synthase, plant growth, photosynthesis, metabolome

## Abstract

*Nicotiana tabacum* solanesyl diphosphate synthase 1 (NtSPS1) is the key enzyme in solanesol biosynthesis. However, changes in the solanesol content, plant growth, photosynthesis, and metabolome of tobacco plants after *NtSPS1* overexpression (OE) have not been previously reported. In the present study, these parameters, as well as photosynthetic gas exchange, chlorophyll content, and chlorophyll fluorescence parameters, were compared between *NtSPS1* OE and wild type (WT) lines of tobacco. As expected, *NtSPS1* OE significantly increased solanesol content in tobacco leaves. Although *NtSPS1* OE significantly increased leaf growth, photosynthesis, and chlorophyll content, the chlorophyll fluorescence parameters in the leaves of the *NtSPS1* OE lines were only slightly higher than those in the WT leaves. Furthermore, *NtSPS1* OE resulted in 64 differential metabolites, including 30 up-regulated and 34 down-regulated metabolites, between the OE and WT leaves. Pathway enrichment analysis of these differential metabolites identified differentially enriched pathways between the OE and WT leaves, e.g., carbon fixation in photosynthetic organisms. The maximum carboxylation rate of RuBisCO and the maximum rate of RuBP regeneration were also elevated in the *NtSPS1* OE line. To our knowledge, this is the first study to confirm the role of *NtSPS1* in solanesol biosynthesis and its possible functional mechanisms in tobacco.

## 1. Introduction

Solanesol, a non-cyclic polyisoprenoid alcohol with nine isoprene units, has antioxidant, anti-inflammatory, antimicrobial, and neuroprotective activities [1,2,3,4]. Recently, the neuroprotective effects of solanesol from behavioural and biochemical perspectives in the intracerebroventricular propionic acid induced experimental model of autism have been studied [4]. Solanesol also serves as an important pharmaceutical intermediate in the chemical synthesis of ubiquinone drugs, including the anticancer agent synergiser N-solanesyl-N,N′-bis(3,4-dimethoxybenzyl) ethylenediamine (SDB), vitamin K2, and coenzyme Q10 [1,5,6]. Among them, SDB can inhibit P-glycoprotein-mediated multidrug resistance and thereby be used to reverse drug resistance of the paclitaxel-resistant cell line (KK47/TX30) [7,8]. The lipid-soluble vitamin K2 has positive effects on the treatment of osteoporosis, promotion of blood clotting, and inhibition of vascular calcification [9]. The lipid-soluble coenzyme Q10 participates in ATP synthesis and oxidative phosphorylation, and it acts as an activator of cellular respiration and cellular metabolism [10]. Moreover, coenzyme Q10 can be used for the treatment of neurodegenerative diseases, renal failure, and cardiovascular diseases [11]. Coenzyme Q10 and solanesyl poly(ethylene glycol) succinate were formulated as micelles to improve the bioavailability of the former in rats [12]. Moreover, solanesyl poly(ethylene glycol) dithiodipropionate and solanesyl thiosalicylic acid micelles were found to serve as efficient drug carriers with synergistic anticancer effects [13,14]. Therefore, the pharmaceutical value of solanesol and its derivatives has been widely confirmed.

Solanesol mainly exists in solanaceous crops, such as tomato (*Solanum lycopersicum*), potato (*Solanum tuberosum*), and tobacco (*Nicotiana tabacum*) [1,5,6,15,16], with *N. tabacum* possessing the highest content [1,5,6]. As solanesol is synthesized in the chloroplast, it mainly accumulates in the leaves and other green tissues [15]. However, its accumulation is also affected by environmental and genetic factors [1,17,18]. Moderately high temperatures [16], pathogen infection [19], long-wavelength/extended irradiation, rare-earth elements, and shade [5] are some of the environmental factors that can lead to increases in the solanesol content of tobacco leaves. In terms of genetic factors, the solanesol content in potato leaves was found to be controlled by quantitative trait genes [18], and that in tobacco leaves was found to be determined by both polygenes and major genes, but the major genes were dominant [20].

Solanesol is synthesized via the methylerythritol 4-phosphate (MEP) pathway in plastids [5,6,15,16]. The synthesis of solanesyl diphosphate from C5 isopentenyl diphosphate and dimethylallyl diphosphate, and the direct precursors (C10 geranyl diphosphate, C15 farnesyl diphosphate, and C20 geranylgeranyl diphosphate) is catalyzed by solanesyl diphosphate synthase (SPS) and represents a key step in solanesol synthesis (Figure S1) [6]. To date, *SPS* genes have been identified in *Arabidopsis thaliana* [21], *Oryza sativa* [22], *S. lycopersicum* [23], and *N. tabacum* [24]. Silencing of *AtSPS1* and *AtSPS2* in *A. thaliana* lowered leaf plastoquinone content, thus reducing photosynthesis and inducing photoinhibition [25]. In *O. sativa*, OsSPS1 and OsSPS2 preferentially catalyze the synthesis of ubiquinone-9 and plastoquinone-9 in mitochondria and plastids, respectively [22]. Overexpression (OE) of tomato *SPS* in tobacco significantly increased the plastoquinone content of immature leaves [23]. The expression levels of *NtSPS1* in different organs of tobacco plants decreased according to this order: leaf > stem > root, which was consistent with the distribution of solanesol in tobacco plants [24]. Studies on *SPS* function have mainly been conducted on *A. thaliana*, rice, tomato, and other model crops, while the function of *NtSPS* genes in tobacco, the plant with the highest solanesol content, has not been reported.

Leaves are the main organs of solanesol accumulation [5,15,17,18] and are also the most active plant tissues for photosynthesis. Chloroplasts are abundant in mesophyll cells, and chloroplasts contain chlorophyll, a green pigment that can absorb light energy [26]. During photosynthesis, plants use light to oxidize water and release oxygen and to reduce carbon dioxide (CO_2_) to synthesize a large number of carbohydrates [27,28]. Photosynthetic gas exchange and chlorophyll fluorescence parameters are the most commonly used phenotypic indicators of plant growth and photosynthesis [29,30]. Moreover, photosynthesis provides the initial substrate and energy for the synthesis of solanesol and other secondary metabolites [16]. Metabolites are the basis of biological phenotypes, which can help us understand biological processes and mechanisms more intuitively and effectively [31]. To date, the changes in solanesol content, plant growth, photosynthesis, and the metabolome of tobacco leaves after *NtSPS1* OE have not been reported. Thus, we compared the differences in solanesol content, plant growth, photosynthetic gas exchange, chlorophyll content, chlorophyll fluorescence parameters, and the metabolome between *NtSPS1* OE and wild type (WT) lines of tobacco plants. The purpose of this study was to analyze the role of *NtSPS1* in solanesol biosynthesis and its possible functional mechanisms to provide a theoretical basis for the regulation of solanesol content in tobacco leaves by means of genetic engineering.

## 2. Results

### 2.1. Total Solanesol Content and NtSPS1 Expression Levels in the Leaves of NtSPS1 OE and WT Tobacco Plants

**Figure 1:**
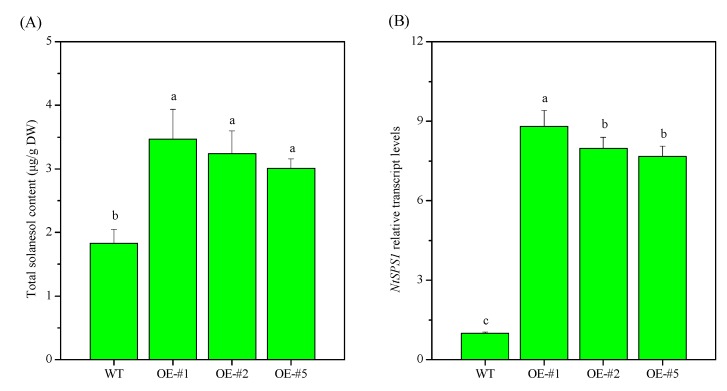
Identification of *NtSPS1* overexpression (OE) tobacco lines. Total solanesol content (**A**) and relative *NtSPS1* transcript levels (**B**) in the leaves of three *NtSPS1* OE and wild type (WT) tobacco plants. Different letters on the columns indicate *p* < 0.05. DW, dry weight.

Eighteen *NtSPS1* transgenic plants were obtained, 16 of which were finally confirmed to be positive (Figure S2). Of these 16 independently derived transgenic lines, five showed *NtSPS1* transcript levels that were >600% WT levels, and the three lines (OE-#1, OE-#2, and OE-#5) with the greatest OE of *NtSPS1* transcripts were investigated further in this study (Figure 1). The total solanesol content in the leaves of OE-#1, OE-#2, and OE-#5, was 89.62%, 77.05%, and 64.48% higher than that in the WT, respectively (Figure 1A), and the relative *NtSPS1* transcript levels in the leaves were 8.80, 7.97, and 7.68 times those of the WT, respectively (Figure 1B). Moreover, the relative *NtSPS1* transcript levels in the leaves of OE-#1 were significantly higher than those in the leaves of OE-#2 and OE-#5 (*p* < 0.05). Thus, OE-#1 was selected as the representative *NtSPS1* OE line for further examination.

**Figure 2:**
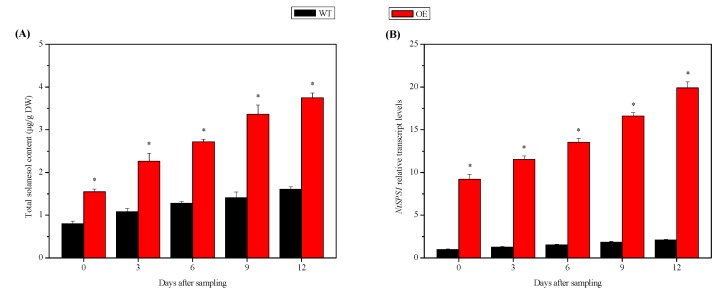
Total solanesol content (**A**) and relative *NtSPS1* transcript levels (**B**) in the leaves of *NtSPS1* overexpression (OE) and wild type (WT) tobacco plants. An asterisk (*) indicates *p* < 0.05 versus the corresponding WT value. DW, dry weight.

We measured the total solanesol content and relative *NtSPS1* transcript levels of the OE and WT tobacco leaves 0–12 days after sampling (DAS). As the leaves grew, their total solanesol content in the *NtSPS1* OE and WT tobacco leaves increased from 0 to 12 DAS (Figure 2A). At 0, 3, 6, 9, and 12 DAS, the total solanesol content in the leaves of the *NtSPS1* OE tobacco plants increased by 93.75%, 109.26%, 112.50%, 138.30%, and 132.92%, respectively, and these contents were significantly higher than those in the WT tobacco leaves (*p* < 0.05) (Figure 2A). At 0, 3, 6, 9, and 12 DAS, the relative *NtSPS1* transcript levels in the leaves of the *NtSPS1* OE tobacco leaves increased 8.21, 8.06, 7.90, 8.02, and 8.52 times, respectively, and were significantly higher than the corresponding levels in the WT tobacco leaves (*p* < 0.05) (Figure 2B). Thus, *NtSPS1* OE significantly increased total solanesol content in the tobacco leaves. 

### 2.2. Growth of the NtSPS1 OE and WT Tobacco Plants

**Figure 3:**
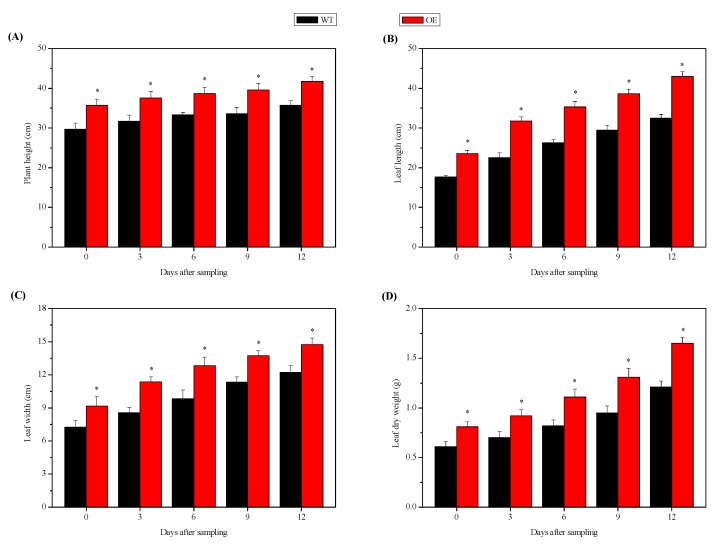
Plant height (**A**), leaf length (**B**), leaf width (**C**), and leaf dry weight (**D**) in the *NtSPS1* overexpression (OE) and wild type (WT) tobacco plants. An asterisk (*) indicates *p* < 0.05 versus the corresponding WT value.

To determine the effects of *NtSPS1* OE on growth of the tobacco plants, we measured the plant height, leaf length, leaf width, and leaf dry weight of the *NtSPS1* OE and WT tobacco plants 0–12 DAS. At 0, 3, 6, 9, and 12 DAS, the plant heights of the *NtSPS1* OE tobacco plants increased by 20.20%, 18.61%, 16.22%, 17.86%, and 16.81%, respectively, and these heights were significantly higher than those of the WT plants (*p* < 0.05) (Figure 3A). At 0, 3, 6, 9, and 12 DAS, the leaf lengths of the *NtSPS1* OE tobacco plants increased by 33.47%, 40.83%, 34.69%, 31.26%, and 32.21%, respectively, values that were significantly higher than those of their WT counterparts (*p* < 0.05) (Figure 3B). Similarly, at 0, 3, 6, 9, and 12 DAS, the leaf widths of the *NtSPS1* OE tobacco plants increased significantly (by 26.13%, 32.71%, 30.52%, 21.18%, and 20.44%, respectively) compared with the corresponding values of the WT plants (*p* < 0.05) (Figure 3C). As expected, the leaf dry weight of the *NtSPS1* OE and WT tobacco plants increased 0–12 DAS (Figure 3D). At 0, 3, 6, 9, and 12 DAS, the leaf dry weights of the *NtSPS1* OE tobacco plants increased by 32.79%, 31.43%, 35.37%, 37.89%, and 36.36%, respectively, and these weights were significantly higher than those of the WT plants (*p* < 0.05) (Figure 3D). Thus, *NtSPS1* OE significantly promoted the growth of tobacco plants and leaves, as reflected by the increased plant height, leaf length, leaf width, and leaf dry weight of the *NtSPS1* OE tobacco plants.

### 2.3. Photosynthetic Gas Exchange in the Leaves of NtSPS1 OE and WT Tobacco Plants

**Figure 4:**
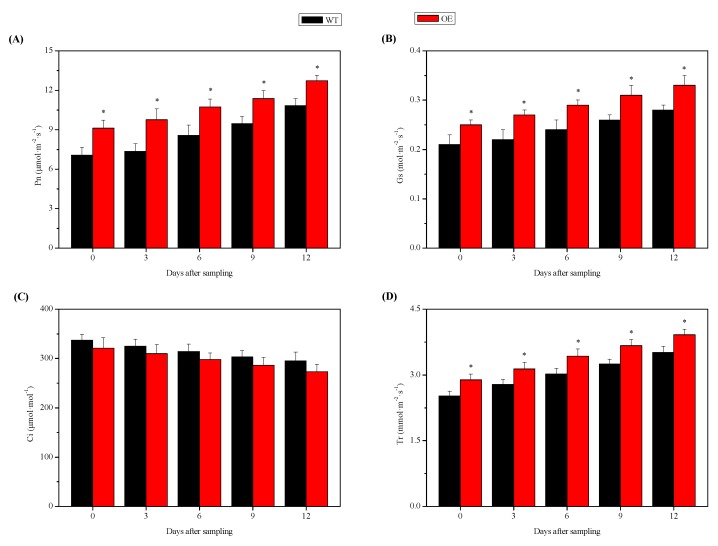
Net photosynthetic rate (Pn, **A**), stomatal conductance (Gs, **B**), intercellular CO_2_ concentration (Ci, **C**), and transpiration rate (Tr, **D**) in the leaves of *NtSPS1* overexpression (OE) and wild type (WT) tobacco plants. An asterisk (*) indicates *p* < 0.05 versus the corresponding WT value.

To determine the effects of *NtSPS1* OE on the photosynthetic gas exchange of tobacco leaves, we measured the net photosynthetic rate (Pn), stomatal conductance (Gs), intercellular CO_2_ concentration (Ci), and transpiration rate (Tr) in the leaves of the *NtSPS1* OE and WT tobacco plants with a portable open gas exchange system. At 0, 3, 6, 9, and 12 DAS, the Pn of the *NtSPS1* OE tobacco leaves increased by 29.14%, 32.74%, 25.20%, 20.19%, and 17.54%, respectively, and these values were significantly higher than those of the WT tobacco leaves (*p* < 0.05) (Figure 4A). At 0, 3, 6, 9, and 12 DAS, the Gs of the *NtSPS1* OE tobacco leaves increased by 19.05%, 22.73%, 20.83%, 19.23%, and 17.86%, respectively, and these values were also significantly higher than those of the WT tobacco leaves (*p* < 0.05) (Figure 4B). However, at 0, 3, 6, 9, and 12 DAS, the Ci of the WT tobacco leaves increased by 4.98%, 4.84%, 5.37%, 5.94%, and 8.06%, respectively, and these values were slightly, but not significantly, higher than those of the *NtSPS1* OE leaves (*p* > 0.05) (Figure 4C). At 0, 3, 6, 9, and 12 DAS, the Tr of the *NtSPS1* OE leaves increased by 14.68%, 12.95%, 13.58%, 12.92%, and 11.68%, respectively, and these values were significantly higher than those of the WT tobacco leaves (*p* < 0.05) (Figure 4D). Thus, *NtSPS1* OE significantly enhanced photosynthesis, as reflected by the increased Pn, Gs, and Tr in the *NtSPS1* OE tobacco leaves.

### 2.4. Chlorophyll Content in the Leaves of NtSPS1 OE and WT Tobacco Plants

**Figure 5:**
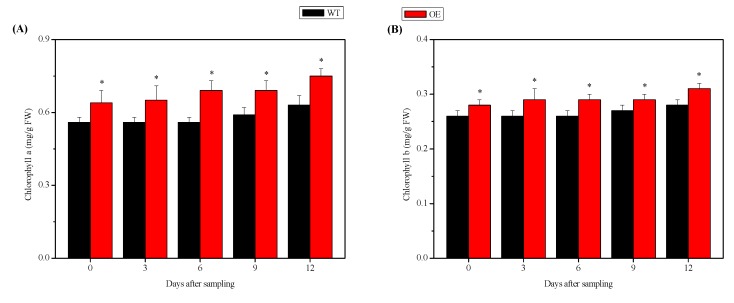
Chlorophyll *a* (**A**) and chlorophyll *b* (**B**) content in the leaves of *NtSPS1* overexpression (OE) and wild type (WT) tobacco plants. An asterisk (*) indicates *p* < 0.05 versus the corresponding WT value.

To determine the effects of *NtSPS1* OE on the chlorophyll content of tobacco leaves, we measured chlorophyll *a* and chlorophyll *b* content in the leaves of *NtSPS1* OE and WT tobacco plants with a UV-2700 UV-VIS spectrophotometer. At 0, 3, 6, 9, and 12 DAS, the chlorophyll *a* content in the *NtSPS1* OE tobacco leaves increased by 14.29%, 16.07%, 23.21%, 16.95%, and 19.05%, respectively, and these values were significantly higher than those in the WT tobacco leaves (*p* < 0.05) (Figure 5A). At 0, 3, 6, 9, and 12 DAS, the chlorophyll *b* content in the *NtSPS1* OE tobacco leaves increased by 7.69%, 11.54%, 11.54%, 7.41%, and 10.71%, respectively, and these values were also significantly higher than those in the WT tobacco leaves (*p* < 0.05) (Figure 5B). Thus, *NtSPS1* OE significantly increased chlorophyll content, as reflected by the increased chlorophyll *a* and chlorophyll *b* levels in the *NtSPS1* OE tobacco leaves.

### 2.5. Chlorophyll Fluorescence Parameters in the Leaves of NtSPS1 OE and WT Tobacco Plants

**Figure 6:**
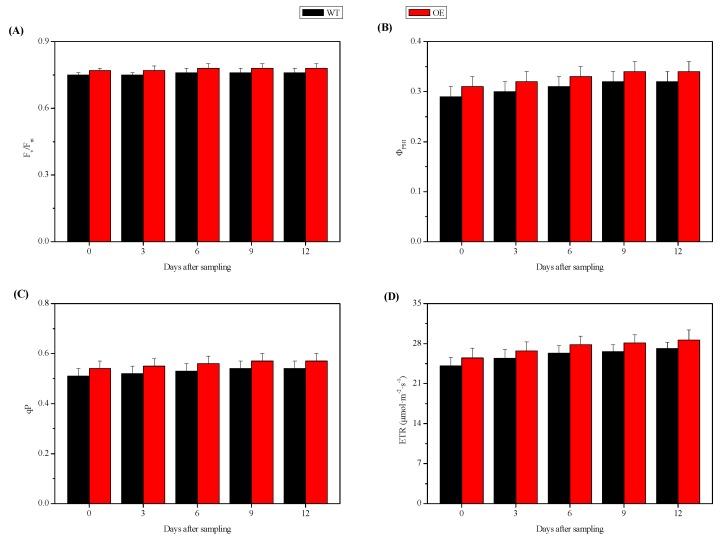
Maximum quantum efficiency of photosystem II in the dark-adapted leaves (F_v_/F_m_, (**A**)), quantum efficiency of photosystem II under light conditions (Φ_PSII_, (**B**)), photochemical quenching (qP, (**C**)), and electron transport rate (ETR, (**D**)) in the leaves of the *NtSPS1* overexpression (OE) and wild type (WT) tobacco plants.

To determine the effects of *NtSPS1* OE on the chlorophyll fluorescence parameters of tobacco leaves, we measured the maximum quantum efficiency of photosystem II (PSII) in dark-adapted leaves (F_v_/F_m_), quantum efficiency of PSII under light conditions (Φ_PSII_), photochemical quenching (qP), and electron transport rate (ETR) in the leaves of the *NtSPS1* OE and WT tobacco plants with an Imaging-PAM-M series chlorophyll fluorometer. At 0, 3, 6, 9, and 12 DAS, the F_v_/F_m_ of the *NtSPS1* OE tobacco leaves increased by 2.67%, 2.67%, 2.63%, 2.63%, and 2.63%, respectively (Figure 6A), and the Φ_PSII_ increased by 6.90%, 6.67%, 6.45%, 6.25%, and 6.25%, respectively (Figure 6B), all slightly higher than the corresponding values in the WT tobacco leaves (*p* > 0.05). Furthermore, at 0, 3, 6, 9, and 12 DAS, the qP of the *NtSPS1* OE tobacco leaves increased by 5.88%, 5.77%, 5.66%, 5.56%, and 5.56%, respectively, (Figure 6C), and the ETR increased by 5.81%, 5.12%, 5.70%, 5.64%, and 5.54%, respectively (Figure 6D), all slightly higher than the corresponding values in the WT tobacco leaves (*p* > 0.05). Thus, *NtSPS1* OE slightly increased chlorophyll fluorescence parameters in tobacco leaves, as reflected by slightly increased F_v_/F_m_, Φ_PSII_, qP, and ETR in the *NtSPS1* OE tobacco leaves, although these differences were not statistically significant.

### 2.6. Leaf Metabolome of the NtSPS1 OE and WT Tobacco Plants

**Figure 7:**
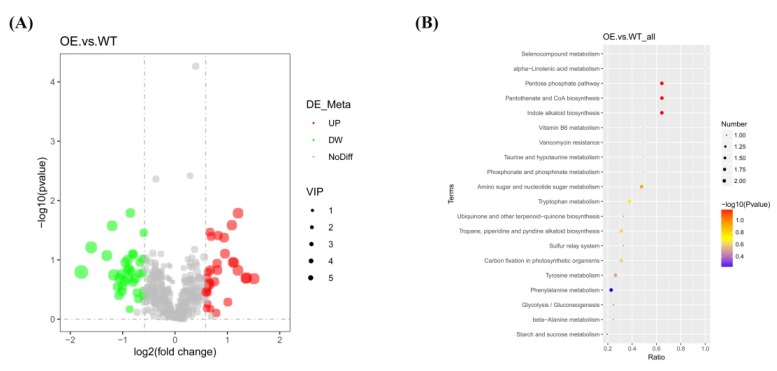
Differential metabolite volcano plot (**A**) and Kyoto Encyclopedia of Genes and Genomes (KEGG) enrichment bubble chart (**B**) between the leaves of the *NtSPS1* overexpression (OE) and wild type (WT) tobacco plants. In part A, the abscissa indicates the expression change (log2 fold change) in the metabolites in the different groups and the ordinate indicates differences in the significance levels (−log10 (*p*-value)). Each point in the volcano plot represents a metabolite and the size of the point indicates the variable importance in project (VIP) value. Significantly up-regulated metabolites are represented by red points, and significantly down-regulated metabolites are represented by green points. In part B, the abscissa indicates the number of differential metabolites in the corresponding metabolic pathway/the number of total metabolites identified in the pathway. The larger the value is, the higher the number of differential metabolites in the pathway. The color of the points represents the *p*-value of hypergeometric test. The smaller the value is, the more reliable and statistically significant the test is. The size of the point represents the number of differential metabolites in the corresponding pathway.

In the present study, 477 metabolites were identified in the *NtSPS1* OE and WT tobacco leaves with a QTRAP^®^ 6500+ mass spectrometer (AB SCIEX, Framingham, MA, USA), comprising two alcohols and polyols, 11 alkaloids and their derivatives, 74 amino acids and their derivatives, six benzene and substituted derivatives, six benzoic acid and substituted derivatives, 39 carbohydrates, one choline, 14 fatty acids, one flavanone, one flavone, one flavone C-glycoside, 15 flavonoids, two flavonols, one free fatty acid, four glycerophospholipids, two hydroxycinnamoyl derivatives, four indole and substituted derivatives, one iridoid glycoside, two ketones, one lactone, 13 lipids and lipid-like molecules, 13 nucleic acid derivatives, 36 nucleotides and their derivatives, 65 organic acids and their derivatives, seven organic oxygen compounds, three organoheterocyclic compounds, one phenolamide, eight phenolamines, one phenolic acid, seven phenol and substituted derivatives, 14 phenylpropanoids and polyketides, one phospholipid, seven phytohormones, 10 polyphenols, eight polyamines, four pyridine and substituted derivatives, two pyrimidines, two quinate and substituted derivatives, two sugar acids and their derivatives, four sugar alcohols, seven TCA cycle intermediates, nine traditional Chinese medicines, six terpenes, six terpenoids, two vitamin derivatives, 16 vitamins, and 35 other metabolites (Table S1). Moreover, 64 metabolites were identified as differential metabolites between the *NtSPS1* OE and WT leaves (Table S2). Compared with the WT tobacco leaves, there were 30 up-regulated and 34 down-regulated metabolites in the *NtSPS1* OE tobacco leaves (Figure 7A; Table S2).

To further clarify the metabolic pathways involved in the synthesis of these differential metabolites, we conducted Kyoto Encyclopedia of Genes and Genomes (KEGG) pathway enrichment analysis, which identified 28 enriched metabolic pathways for the differential metabolites between *NtSPS1* OE and WT tobacco leaves (Table S3). Twenty of the most enriched pathways were selected for further study (Figure 7B). Pathways that encompassed two differential metabolites included “phenylalanine metabolism,” “tyrosine metabolism,” “carbon fixation in photosynthetic organisms,” “tropane, piperidine, and pyridine alkaloid biosynthesis,” “tryptophan metabolism,” “amino sugar and nucleotide sugar metabolism,” “indole alkaloid biosynthesis,” “pantothenate and CoA biosynthesis,” and “pentose phosphate pathway”. The pathways that encompassed one differential metabolite included “starch and sucrose metabolism,” “beta-alanine metabolism,” “glycolysis/gluconeogenesis,” “sulfur relay system,” “ubiquinone and other terpenoid−quinone biosynthesis,” “phosphonate and phosphinate metabolism,” “taurine and hypotaurine metabolism,” “vancomycin resistance,” “vitamin B6 metabolism,” “alpha−linolenic acid metabolism,” and “selenocompound metabolism”. Among these enriched metabolic pathways, the “carbon fixation in photosynthetic organisms” pathway is closely related to photosynthesis.

### 2.7. Maximum Carboxylation Rate of RuBisCO and Maximum Rates of RuBP Regeneration in Leaves of the NtSPS1 OE and WT Tobacco Plants

To determine the effects of *NtSPS1* OE on carbon fixation in tobacco leaves, we used a mathematical model to calculate the maximum carboxylation rate of RuBisCO (Vc,max) and maximum rates of RuBP regeneration (Jmax) in the leaves of the *NtSPS1* OE and WT tobacco plants. Both Vc,max and Jmax increased significantly in the *NtSPS1* OE leaves (by 17.20% and 16.06%, respectively), compared with the corresponding value in the WT tobacco leaves (*p* < 0.05).

## 3. Discussion

### 3.1. NtSPS1 OE Enhances Solanesol Accumulation in Tobacco Leaves

The solanesol content in tobacco leaves is influenced by both environmental and genetic factors [17,18], but genetic factors exert a considerable influence [1]. To identify solanesol-rich tobacco varieties, Xiang et al. [20] determined the total solanesol content in the leaves of 168 flue-cured tobacco sources from various years and regions in China and found that their solanesol content was between 0.70% and 4.13%. To analyze the influence of solanesol biosynthetic genes on solanesol accumulation in tobacco, Gai et al. [32] measured solanesol content in the leaves, stems, and roots and quantified solanesol biosynthetic gene expression in the tobacco cultivars “Zhongyan90,” which has low solanesol content, and “Hongda,” which has high solanesol content. Their results indicated that the solanesol biosynthetic genes in both “Zhongyan90” and “Hongda” may regulate solanesol content through synergistic effects. In addition, transient OE of genes from the mevalonic acid and MEP pathways significantly increased solanesol content in the leaves of *Nicotiana benthamiana* [18]. Similarly, overexpression of the 1-deoxy-d-xylulose-5-phosphate reductoisomerase gene in chloroplasts increased the solanesol content in tobacco leaves [33]. Overexpressing the key enzyme gene of solanesol biosynthesis, *NtSPS1*, significantly increased the total solanesol content in tobacco leaves under day/night temperatures of 30/24 °C (Figure 1 and Figure 2). In our previous study, moderately high temperatures (day/night temperatures of 30/24 °C) resulted in significantly higher solanesol content and *NtSPS1* expression than normal temperatures (day/night temperatures of 22/16 °C), suggesting that the increased expression of *NtSPS1* is related to the increase in solanesol content under moderately high temperatures [16]. Solanesol is synthesized in chloroplasts [5,6,15,16]. We cannot exclude the possibility that the increase in total solanesol content in the *NtSPS1* OE tobacco leaves was related to their increased chlorophyll content. Regardless, to the best of our knowledge, the present study is the first to confirm the role of *NtSPS1* in solanesol biosynthesis in tobacco. 

### 3.2. NtSPS1 OE Enhances Tobacco Plant Growth and Photosynthesis

The leaf is the organ with the highest solanesol accumulation [5,15]. Thus, leaf biomass has a significant effect on the final yield of solanesol. The present study showed that *NtSPS1* OE significantly increased the length, width, and dry weight of tobacco leaves (Figure 3). Similarly, tobacco plants overexpressing H-protein showed higher leaf area and leaf dry weight than WT tobacco plants [34]. In the present study, *NtSPS1* OE increased the solanesol content in tobacco leaves (Figure 1 and Figure 2) and also increased the biomass of tobacco leaves, which thus greatly increased the final yield of solanesol from the entire tobacco plant. Similarly, OE of the *small auxin-up RNA* gene significantly increased the biomass and content of tropine alkaloids in *Atropa belladonna* [35].

The augmenting effect of *NtSPS1* OE on tobacco leaf biomass is related to an increased Pn of the leaves (Figure 4). Moreover, *NtSPS1* OE may enhance Pn by increasing Gs or by affecting non-stomatal biochemical processes. The present study showed that the Gs of the *NtSPS1* OE leaves was significantly higher than that of the WT tobacco leaves (Figure 4). The increase in Tr in the *NtSPS1* OE tobacco leaves was possibly related to the increase in Gs and corresponded with the stomatal aperture [36]. In the present study, enhanced Gs in the *NtSPS1* OE tobacco leaves was beneficial for CO_2_ movement in the stomatal cavity. In addition, the increase in Pn caused by *NtSPS1* OE was probably mediated through non-stomatal biochemical processes, such as elevations in chlorophyll content (Figure 5) and ETR (Figure 6). Similarly, OE of the cotton *FLOWERING LOCUS T* (*FT*)-like gene [37] or *Medicago sativa glutamate-semialdehyde aminotransferase* gene [38] led to increased chlorophyll content and photosynthesis efficiency in tobacco leaves. In rice, OE of the *A. thaliana* NAD kinase gene caused increased ETR and CO_2_ assimilation rates [39]. In *A. thaliana*, the OE of *AtSPS1* can lead to the accumulation of plastoquinone-9 and its derivative plastochromanol-8 in the leaves and can reduce lipid peroxidation and PSII photoinhibition under excess light [40]. In *Salvia miltiorrhiza*, the OE of polyprenyl diphosphate synthase 1 also significantly increased the content of plastoquinone-9, which is the main carrier of photosynthetic electrons [41]. In our previous study, moderate/high temperatures resulted in significantly higher Pn and *NtSPS1* expression in tobacco leaves than normal temperatures [16]. The present study confirmed that *NtSPS1* OE enhances the biomass and photosynthesis of tobacco leaves. Moreover, the increase in Pn in the *NtSPS1* OE tobacco leaves augments initial substrate levels and energy for solanesol biosynthesis.

### 3.3. Effects of NtSPS1 OE on the Metabolome of Tobacco Leaves

In the present study, *NtSPS1* OE resulted in 64 differential metabolites between the *NtSPS1* OE and WT leaves, including 30 up-regulated and 34 down-regulated metabolites (Figure 7A). In *Brassica napus*, OE of the lipid transfer protein BraLTP2 altered the accumulation of secondary metabolites in leaves, including 43 up-regulated and 30 down-regulated secondary metabolites [42]. The present study indicated that *NtSPS1* OE not only enhanced solanesol accumulation in tobacco leaves but also altered many metabolic pathways in the leaves (Figure 7B). Similarly, amorpha-4,11-diene synthase overexpression not only affects artemisinin biosynthesis but also affects the whole metabolic network of terpenoids in *Artemisia annua* [43]. The KEGG pathway enrichment analysis indicated that 28 metabolic pathways were enriched to produce the differential metabolites between *NtSPS1* OE and WT tobacco leaves. Among these metabolic pathways, carbon fixation in photosynthetic organisms [44] is closely related to plant photosynthesis. Moreover, *NtSPS1* OE may enhance carbon fixation by increasing ribose 5-phosphate content (Tables S2 and S3), which affects CO_2_ fixation in chloroplasts [45]. The increased CO_2_ fixation rate could result from alterations in only part of the carbon reduction cycle utilizing ATP from the photochemical reactions to convert ribose 5-phosphate to ribose 5-diphosphate (the carboxylation reaction substrate) [46]. Therefore, the enhanced photosynthesis by *NtSPS1* OE may be related to the ability of *NtSPS1* OE to promote carbon fixation (Vc,max and Jmax) in tobacco leaves (Table 1). Except for “carbon fixation in photosynthetic organisms,” the pathways that encompassed two differential metabolites between the *NtSPS1* OE and WT tobacco leaves included “phenylalanine metabolism”; “tyrosine metabolism”; “tropane, piperidine, and pyridine alkaloid biosynthesis”; “tryptophan metabolism”; “amino sugar and nucleotide sugar metabolism”; “indole alkaloid biosynthesis”; “pantothenate and CoA biosynthesis”; and “pentose phosphate pathway” (Figure 7B). Thus, KEGG pathway enrichment analysis of the differential metabolites identified enriched pathways between the *NtSPS1* OE and WT tobacco leaves.

## 4. Materials and Methods

### 4.1. Plant Materials

WT tobacco (*N. tabacum* “Hongda”) seeds were obtained from the National Infrastructure for Crop Germplasm Resources (Tobacco, Qingdao, China). Construction of the *NtSPS1* OE tobacco plants was carried out as indicated below.

#### 4.1.1. Construction of the NtSPS1 OE Vector

**Figure 8:**
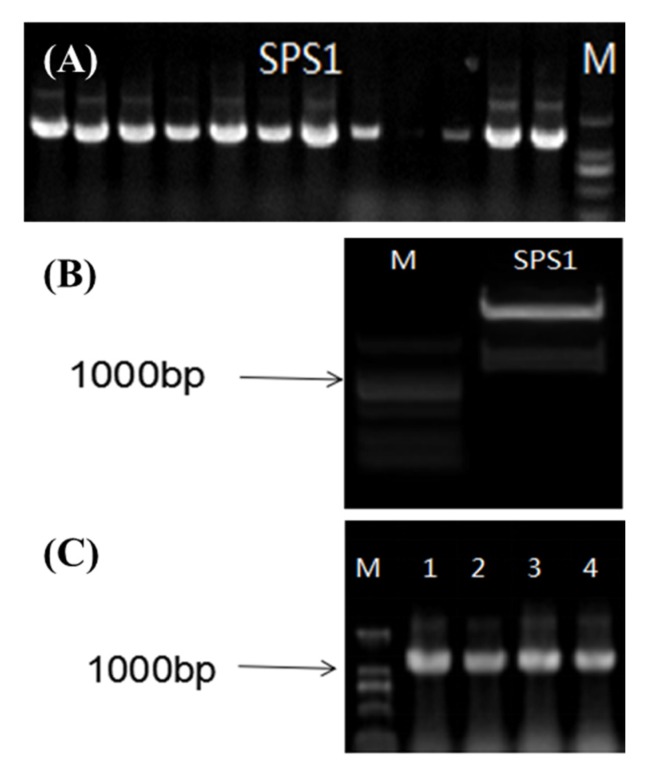
PCR screening of *Escherichia coli* clones transformed with the *pCHF3-NtSPS1* plasmid (**A**), agarose gel electrophoresis of *NtSPS1* gene excised from a positive clone by *Sma*I/*Sal*I double digestion (**B**), and PCR screening of bacterial clones transformed with *NtSPS1* ligated into the OE vector (**C**). M—marker.

*NtSPS1* was cloned according to the sequence obtained from a BLAST search against the Gene-Space Sequence Reads from the China Tobacco Genome database, as described previously [24]. Considering the restriction sites of the OE vector *pCHF3* and the *NtSPS1* fragment, the restriction site *Sma*I (CCCGGG) was added to the upstream primer for *NtSPS1* and the restriction site *Sal*I (GTCGAC) was added to the downstream primer for *NtSPS1*. The sequences of the primers with the adapters were 5′-TCCCCCGGGATGATGTCTGTGACTTGCCATAATCTTGAG-3′ (upstream primer) and 5′-CGCGTCGACCTATTCAATTCTCTCCAGATTATACTTCAC-3′ (downstream primer), and the Tm of the primers was 60 °C. The high-fidelity enzyme KD-Plus was used to amplify the target gene, and the products were subjected to agarose gel electrophoresis. Through a gel documentation system, the band corresponding to the size (~1200 bp) of the target fragment was subjected to gel extraction. The fragment was ligated to the cloning vector and transformed into competent *Escherichia coli*. Colonies containing the target fragments were selected via antibiotic resistance and PCR screening (Figure 8A—most of the selected clones were positive) and were then verified by sequencing after propagation. Thus, the PCR-amplified *NtSPS1* coding sequence was successfully ligated into the *pCHF3* binary vector and cloned in *E. coli*.

Positive colonies with the expected sequencing results were further propagated, and positive plasmids were isolated from these colonies. The positive plasmids and OE vector were subjected to *Sma*I/*Sal*I double digestion using the following digestion system at 37 °C for 2 h. The double-digested fragment of the *NtSPS1* gene is shown in Figure 8B. The digestion products from the positive plasmids and the OE vector were purified by agarose gel electrophoresis. The recovered products were ligated overnight at 16 °C, with a volume ratio of insert:vector:T4 DNA Ligase:T4 DNA ligase buffer of 5:3:1:1. The ligation mixture was subjected to transformation in *E. coli*. The PCR screening results of the *NtSPS1* recombinants are shown in Figure 8C. The sequence-verified OE constructs obtained from the positive colonies were transformed into *Agrobacterium tumefaciens* for subsequent plant transformation.

#### 4.1.2. *A. tumefaciens*–Mediated Genetic Transformation

Fifty microliters of a stored *A. tumefaciens* culture was transferred to a 50 mL small conical flask containing LB medium with kanamycin and rifampicin. The flask was cultured in an incubator at 28 °C and 200 rpm constant shaking for approximately 12 h, until the color of the bacterial suspension turned from brownish red to dark yellow and its optical density (OD) reached approximately 0.6. The bacterial suspension was then transferred to a 50 mL centrifuge tube and centrifuged at 15,000× *g* for 10 min. After removing the supernatant, the bacterial pellet was resuspended in sterile water and, after adjusting the OD to approximately 0.6, acetosyringone was added (final concentration 20 mg/L). The bacterial suspension was then transferred to a large sterilized conical flask to prepare for the transfection.

Tobacco leaves were transferred with tweezers from a tissue culture flask to sterilized filter paper. The edges and main veins of the leaves were removed with scissors, and the leaves were cut into 1.2 × 1.2 cm pieces. These small pieces were then transferred to the conical flask containing the bacterial suspension, shaken, and soaked for 8–10 min. After this period, the small pieces were spread on co-cultivation medium, with their upper surfaces facing down. The culture dish was covered with plastic wrap, placed in a greenhouse, and cultured in the dark for 48–60 h. When the leaves started to differentiate, the differentiated shoots were excised, and transferred onto rooting medium. The rooting status of the seedlings was observed until the seedlings grew to ~5 cm in height, with 3–5 roots each. At this point, the lid of the tissue culture flask was unscrewed, an appropriate amount of sterile water was added, and seedling hardening was performed after cultivation in a greenhouse for approximately three days. The hardened seedlings were then transplanted to pots containing sterilized nutrient soil. After transplanting, water was provided as required to promote root system growth and the transgenic plants were later obtained.

#### 4.1.3. Molecular Detection of the Transgenic Plants

The transgenic-positive plants contained the *Pchf3* vector with the 35S promoter, whereas the non-transgenic plants did not. Therefore, a forward primer annealing in the 35S promoter sequence and a reverse primer annealing in the *NtSPS1* sequence (downstream primer 5′-AAAGTCGACCTATTCAATTCTCTCCAGATTATACTTCAC-3′) were used for positive verification of the T0 *NtSPS1* OE plants. The size of the amplified fragment was approximately 1400 bp. DNA from the transgenic tobacco leaves was used as template for PCR amplification. The plasmid DNA of the OE vector was used as the positive control, and DNA from non-transgenic tobacco leaves was used as the negative control. Agarose gel electrophoresis of the PCR products was performed to determine whether the transgenic plants were positive. 

### 4.2. Plant Growth Conditions

Seeds of the *NtSPS1* OE and WT tobacco plants were germinated in a growth substrate that contained a mixture of vermiculite and peat (1:2, v/v). After germination, the plants (~15 cm height) were transferred to pots containing the above growth substrate. After eight weeks, the plants were moved to an illumination incubator (MGC-250; Yihengyiqi, Suzhou, Jiangsu, China) under day/night temperatures of 30/24 °C and a 12-h photoperiod. After four weeks in the illumination incubator, tobacco leaves were sampled to determine photosynthetic gas exchange and chlorophyll fluorescence. Leaf samples were harvested from the fifth fully expanded leaf from the top of the plant at 0, 3, 6, 9, and 12 DAS. The collected tobacco samples were mainly used for assessing solanesol and chlorophyll content and the metabolome.

### 4.3. Analysis of Total Solanesol Content

Extraction of total solanesol from tobacco leaves of the *NtSPS1* OE and WT tobacco plants was performed according to the procedure described by Yan et al. [16]. The tobacco leaves were dried to constant weight using a freeze-dryer (Alpha 1–2 LD Plus; Christ, Osterode, Lower Saxony, Germany), ground, and sifted through a 40-mesh sieve. Portions (0.2 g) of the powdered samples were transferred to individual 20-mL centrifuge tubes with stoppers, and 1 mL 1 M NaOH (diluted in ethanol) and 5 mL hexane were added. Ultrasonic extraction was performed at 50 °C for 30 min, and 8 mL distilled water was added. After centrifugation at 3000× *g* for 10 min, 0.5 mL of the supernatants was sampled, diluted with 4.5 mL methanol in brown volumetric flasks, and filtered through a 0.2-μm membrane. Total solanesol content was measured using ultra-high performance liquid chromatography (ACQUITY UPLC H-Class; Waters, Milford, MA, USA) with an Atlantis T3-C_18_ column (4.6 × 150 mm, 3 μm; Waters) that was maintained at 35 °C. A 50:50 (v/v) methanol-acetonitrile solution was used as the mobile phase at a flow rate of 1.0 mL/min, and a diode array detector was used for detection at 208 nm. 

### 4.4. Quantitative Reverse Transcription PCR of NtSPS1

Quantitative reverse transcription PCR (qRT-PCR) of *NtSPS1* gene expression from the leaves of the *NtSPS1* OE and WT tobacco plants was performed according to the procedure described by Yan et al. [16]. Gene-specific primer pairs were designed to detect *NtSPS1* (upstream primer: 5′-CATTCCAAATATGAGATGCGTTGT-3′; downstream primer: 5′-TGTGGACTTGGGAGAGGACT-3′). Two fragments of the constitutively expressed *Ntactin* gene were amplified as references using the gene-specific upstream and downstream primers 5′-TGTCTGTGACTTGCCATAA-3′ and 5′-CATTGAATCCTCCTCTACTT-3′, respectively. The qRT-PCR was performed using an ABI 7500 Real-time system (Applied Biosystems, Foster City, CA, USA).

### 4.5. Measurements of Plant Growth

The height, leaf length, leaf width, and leaf dry weight of the *NtSPS1* OE and WT tobacco plants were determined at five stages (0, 3, 6, 9, and 12 DAS) with three replicates. The leaf dry weight was measured after freeze-drying for three days (Alpha 1–2 LD Plus; Christ, Osterode am Harz, Germany).

### 4.6. Photosynthetic Gas Exchange Measurements

Photosynthetic gas exchange was measured with a portable open gas exchange system (Li-6400, Li-Cor, Inc., Lincoln, NE, USA) according to the procedure described by Yan et al. [30]. The fifth fully expanded leaf from the top of the *NtSPS1* OE and WT tobacco plants was used at five stages (0, 3, 6, 9, and 12 DAS). All measurements were performed at a CO_2_ concentration of 400 μmol·mol^−1^ with a photosynthetic photon flux density of 1000 μmol·m^−2^·s^1^. The CO_2_ response curve of photosynthesis at 12 DAS was measured according to the procedure described by Yan et al. [30], and Vc,max and Jmax were calculated according to the procedure described by Ethier and Livingston [47].

### 4.7. Determination of Chlorophyll Content

Chlorophyll was extracted from the leaves of the *NtSPS1* OE and WT tobacco plants at five stages (0, 3, 6, 9, and 12 DAS) according to the procedure described by Yan et al. [29]. The absorbance of the filtered extracts at 645 nm (D_645_) and 663 nm (D_663_) was determined using a UV-2700 UV-VIS spectrophotometer (Shimadzu, Tokyo, Japan). Chlorophyll *a* (C_A_) and chlorophyll *b* (C_B_) content was then calculated using the following equations: C_A_ = 0.125 × (13.7 × (D_663_) − 5.76 × (D_645_)) and C_B_ = 0.125 × (25.8 × (D_645_) − 7.6 × (D_663_)).

### 4.8. Measurements of Chlorophyll Fluorescence Parameters

Chlorophyll fluorescence parameters of the *NtSPS1* OE and WT tobacco leaves were measured at five stages (0, 3, 6, 9, and 12 DAS) with an Imaging-PAM-M series chlorophyll fluorometer (Heinz Walz, Effeltrich, Germany) according to the procedure described by Yan et al. [29,30]. The F_v_/F_m_, Φ_PSII_, qP, and ETR were exported using Imaging-WIN software.

### 4.9. Targeted Metabolomics

#### 4.9.1. Metabolite Extraction

Tobacco leaves collected at 12 DAS were used to analyze the leaf metabolome. The leaves (100 mg) were ground in liquid nitrogen, and the homogenate was resuspended in 500 μL of prechilled 80% methanol and 0.1% formic acid by vortexing. The samples were incubated on ice for 5 min and then centrifuged at 15,000× *g* and 4 °C for 10 min. The supernatant was diluted to 53% methanol with LC-MS grade water. The samples were subsequently transferred to a clean Eppendorf tube and centrifuged at 15,000× *g* and 4 °C for 20 min. Finally, the supernatant was injected into the LC-MS/MS system.

Equal volumes of each experimental sample were pooled as the quality control sample. The blank sample was an aqueous solution of 53% methanol containing 0.1% formic acid. The pretreatment process was the same as that for the experimental samples. The liquid sample (100 μL) and prechilled methanol (400 μL) were mixed by vortexing. The cell sample (50 μL) and prechilled 80% methanol (200 μL) were mixed by vortexing and then sonicated for 6 min. This step was repeated once before performing the steps described above.

#### 4.9.2. HPLC-MS/MS Analysis

**Positive Ion Mode:** LC-MS/MS analyses were performed using an Exion LC™ AD system coupled with a QTRAP^®^ 6500+ mass spectrometer (both from AB SCIEX, Framingham, MA, USA). The samples were injected into a BEH C8 column (100 × 2.1 mm, 1.9 μm) using a 30 min linear gradient at a flow rate of 0.35 mL/min for positive polarity mode. The eluents were eluent A (0.1% formic acid-water) and eluent B (0.1% formic acid-acetonitrile). The solvent gradient was as follows: 5% B, 1.0 min; 5–100% B, 24.0 min; 100% B, 28.0 min; 100–5% B, 28.1 min; 5% B, 30 min. The QTRAP^®^ 6500+ mass spectrometer was operated in positive polarity mode with the curtain gas at 35 psi, collision gas at medium, ion spray voltage of 5500 V, temperature of 500 °C, ion source gas of 1:55, and ion source gas of 2:55.

**Negative Ion Mode:** The samples were injected into an HSS T3 column (100 mm × 2.1 mm) using a 25 min linear gradient at a flow rate of 0.35 mL/min for negative polarity mode. The eluents were eluent A (0.1% formic acid-water) and eluent B (0.1% formic acid-acetonitrile). The solvent gradient was as follows: 2% B, 1.0 min; 2–100% B, 18.0 min; 100% B, 22.0 min; 100–5% B, 22.1 min; 5% B, 25 min. The QTRAP^®^ 6500+ mass spectrometer was operated in negative polarity mode with the curtain gas at 35 psi, collision gas at medium, ion spray voltage of –4500 V, temperature of 500 °C, ion source gas of 1:55, and ion source gas of 2:55.

#### 4.9.3. Metabolite Identification and Quantification

The detection of experimental samples using multiple reaction monitoring was based on an in-house database (Novogene, Beijing, China). The product ion was used for metabolite quantification, and the precursor ion, product ion, retention time, declustering potential, and collision energy were used for metabolite identification. The data files generated by HPLC-MS/MS were processed using SCIEX OS Version 1.4 (AB SCIEX, Framingham, MA, USA) to integrate and correct the peaks. The main parameters were set as follows: minimum peak height, 500; signal/noise ratio, 10; and Gaussian smooth width, 3. The area of each peak indicated the relative content of the corresponding substance.

#### 4.9.4. Data Analyses

Metabolites were annotated using the KEGG database (http://www.genome.jp/kegg/), Human Metabolome Database (http://www.hmdb.ca/), and Lipid maps database (http://www.lipidmaps.org/). We applied univariate analysis (*t*-test) to calculate the statistical significance (*p*-value). The metabolites with the variable importance in project (VIP) > 1.0 and *p*-value < 1.0, and a fold change (FC) ≥ 1.5 or ≤ 0.667, were considered differential metabolites [48,49]. Volcano plots were used to filter the metabolites of interest, based on the Log2 (FC) and -log10 (*p*-value) of the metabolites. The functions of these differential metabolites and metabolic pathways were studied using the KEGG database.

### 4.10. Statistical Analysis

Data are reported as means ± SD. Student’s *t*-tests were used to determine significant differences between the *NtSPS1* OE and WT tobacco plants. Multiple means were compared by analysis of variance followed by Duncan’s multiple range tests. In the present study, *p* < 0.05 was considered significant.

## 5. Conclusions and Future Perspectives

In the present study, *NtSPS1* OE not only significantly increased the solanesol content in tobacco leaves but also significantly increased tobacco leaf growth and, thus, greatly increased the final yields of solanesol. Moreover, *NtSPS1* OE significantly enhanced photosynthesis (as reflected by increased Pn, Gs, and Tr) and chlorophyll content (as reflected by increased chlorophyll *a* and chlorophyll *b* content) in tobacco leaves. However, *NtSPS1* OE only slightly increased chlorophyll fluorescence parameters in the tobacco leaves (as reflected by slightly increased F_v_/F_m_, Φ_PSII_, qP, and ETR). Thus, *NtSPS1* OE may enhance Pn by affecting CO_2_ diffusivity into the leaf (increase in Gs), increased chlorophyll concentration (i.e., energy captured by photosystems), and ETR. *NtSPS1* OE resulted in 64 differential metabolites between the leaves of *NtSPS1* OE and WT tobacco plants, including 30 up-regulated and 34 down-regulated metabolites. KEGG pathway enrichment analysis of these differential metabolites identified enriched pathways between the *NtSPS1* OE and WT tobacco leaves, e.g., carbon fixation in photosynthetic organisms. Notably, *NtSPS1* OE may enhance photosynthesis in tobacco leaves by promoting carbon fixation (Vc,max and Jmax). To the best of our knowledge, this is the first study to confirm the role of *NtSPS1* in tobacco solanesol biosynthesis and how *NtSPS1* OE affects the growth, photosynthesis, and metabolome of tobacco plants.

Compared with normal temperature (day/night temperature 22/16 °C), a moderately high temperature (day/night temperature 30/24 °C) led to a significant increase in the solanesol content and expression of *NtSPS1*, suggesting that *NtSPS1* is related to the significant increase in solanesol content induced by moderately high temperature. We have constructed *NtSPS1* OE vectors and generated the corresponding transgenic tobacco lines. In a future study, we will construct *NtSPS1* gene-knockout vectors and generate the corresponding transgenic tobacco lines, which will be used for further investigation. The solanesol content, physiological characteristics, and cell ultrastructure of the transgenic and control plants will be evaluated under normal and moderately high temperature conditions. Under different temperature conditions, RNA-sequencing will be used to screen differentially expressed genes in the transgenic and control plants and qRT-PCR will be used to validate the differences in key genes of solanesol biosynthesis. The results are expected to reveal the roles and mechanisms of *NtSPS1* in the regulation of solanesol biosynthesis in tobacco induced by moderately high temperatures.

## Figures and Tables

**Table 1:** Maximum carboxylation rate of RuBisCO (Vc,max) and maximum rates of RuBP regeneration (Jmax) in the leaves of *NtSPS1* OE and WT tobacco plants 12 days after sampling. An asterisk (*) indicates *p* < 0.05 versus the corresponding WT value.

	Vc,max (μmol·m^−2^·s^−1^)	Jmax (μmol·m^−2^·s^−1^)
WT	68.6 ± 3.9	153.8 ± 6.4
OE	80.4 ± 4.1 *	178.5 ± 6.8 *

## Supplementary Materials

The following are available online at https://www.mdpi.com/2223-7747/9/4/518/s1, Figure S1: The role of SPS in solanesol biosynthesis. Figure S2: PCR analysis of the transgenic plants carrying the *NtSPS1* gene construct. Table S1: Metabolites identified in tobacco leaves. Table S2: Differential metabolites between the *NtSPS1* overexpression (OE) and wild type (WT) tobacco leaves. Table S3: KEGG pathway enrichment analysis of differential metabolites between the *NtSPS1* overexpression (OE) and wild type (WT) tobacco leaves.
