# Supplementary material for: Effects of NtSPS1 Overexpression on Solanesol Content, Plant Growth, Photosynthesis, and Metabolome of Nicotiana tabacum
Source: Plants (Basel). 2020 Apr 17;9(4):518. doi: 10.3390/plants9040518 (PMC7238068; doi:10.3390/plants9040518)
Supplement: Supplementary file 1 [file plants-09-00518-s001.zip › Supplementary Material/Supplementary Figures.doc]

**Supplementary Figure S1.** The role of SPS in solanesol biosynthesis. SPS catalyses the synthesis of solanesyl diphosphate from the C5 isopentenyl diphosphate and dimethylallyl diphosphate, and the direct precursors (C10 geranyl diphosphate, C15 farnesyl diphosphate, and C20 geranyl geranyl diphosphate). Enzymes involved in this pathway: IPI, isopentenyl diphosphate isomerase; GPS, geranyl diphosphate synthase; FPS, farnesyl diphosphate synthase; GGPPS, geranyl geranyl diphosphate synthase; SPS, solanesyl diphosphate synthase.

**Supplementary Figure S2.** PCR analysis of the transgenic plants carrying the *NtSPS1* gene construct. M: molecular marker. K: Wild type (WT) control, Y: negative control (vector only). Lines #1-18: *NtSPS1*-overexpressing lines. Among these lines #3 and #15 were identified as false positives.
